# *Urtica dioica* attenuates ovalbumin-induced inflammation and lipid peroxidation of lung tissues in rat asthma model

**DOI:** 10.1080/13880209.2017.1310905

**Published:** 2017-04-07

**Authors:** Hanene Zemmouri, Omar Sekiou, Sonda Ammar, Abdelfattah El Feki, Mohamed Bouaziz, Mahfoud Messarah, Amel Boumendjel

**Affiliations:** a Laboratory of Biochemistry and Environmental Toxicology, Department of Biochemistry, Faculty of Sciences, University of Badji Mokhtar, Annaba, Algeria;; b Laboratory of Applied Biochemistry and Microbiology, Department of Biochemistry, Faculty of Sciences, University of Badji Mokhtar, Annaba, Algeria;; c Laboratory of Electrochemistry and Environment, National Engineering School of Sfax, Sfax, Tunisia;; d Faculty of Sciences of Sfax, Laboratory of Animal Ecophysiology, Sfax, Tunisia

**Keywords:** Anti-asthmatic, nettle, polyphenols, airway inflammation, oxidative stress

## Abstract

**Context:** To find bioactive medicinal herbs exerting anti-asthmatic activity, we investigated the effect of an aqueous extract of *Urtica dioica* L. (Urticaceae) leaves (UD), the closest extract to the Algerian traditional use.

**Objective:** In this study, we investigated the *in vivo* anti-asthmatic and antioxidant activities of nettle extract.

**Materials and methods:** Adult male Wistar rats were divided into four groups: Group I: negative control; group II: Ovalbumin sensitized/challenged rats (positive control); group III: received UD extract (1.5 g/kg/day) orally along the experimental protocol; group IV: received UD extract (1.5 g/kg/day) orally along the experimental protocol and sensitized/challenged with ovalbumin. After 25 days, blood and tissue samples were collected for haematological and histopathological analysis, respectively. The oxidative stress parameters were evaluated in the lungs, liver and erythrocytes. Then, correlations between markers of airway inflammation and markers of oxidative stress were explored.

**Results:** UD extract significantly (*p* < 0.01) inhibited eosinophilia increases in BALF (−60%) and the levels of leucocytes (−32.75%) and lymphocytes (−29.22%) in serum, and effectively suppressed inflammatory cells recruitment in the asthmatic rat model. Besides, the lipid peroxidation generated by allergen administration was significantly (*p* < 0.05) diminished by UD treatment in lung tissue (−48.58%). The nettle extract was also investigated for the total phenolic content (30.79 ± 0.96 mg gallic acid/g dry extract) and shows DPPH radical scavenging activity with 152.34 ± 0.37 μg/mL IC_50_ value.

**Conclusions:** The results confirmed that UD administration might be responsible for the protective effects of this extract against airway inflammation.

## Introduction

Asthma is a chronic inflammatory lung disease, characterized by an influx and activation of inflammatory cells. The notion of oxidative stress is found in this type-I hypersensitivity reactions in which the inflammation of the airways is caused by the production of reactive oxygen species (ROS) and secreted by inflammatory cells (Dworsky 2000). These ROS can directly be responsible for lung injury and contraction of the bronchial muscles, participating in the typical bronchial obstruction in asthma.

Thus, the imbalance between defence and free-radical production systems causes lesions at the level of the body cells. Therefore, several treatments to reduce the phenomenon of oxidative stress have been considered, including plant extracts used in diseases of the respiratory system (Lee et al. 2010a, 2010b; Tiwari et al. 2014; You et al. 2014).

The growing interest in natural antioxidants as alternative to synthetic products, to the adverse secondary effects, has led to the recovery of a large number of readily available plant resources that are inexpensive, but of some interest.

The nettle, *Urtica dioica* L. (Urticaceae), is a medicinal herb, several clinical studies have shown the efficacy of external treatment of nettle leaves on joint pain (Randall et al. 2000) and their internal use in arthritis, rheumatism (Chrubasik et al. 1997) and allergic rhinitis (Mittman 1990). Several other studies have confirmed the anti-inflammatory (Kavalali 2003) and immunomodulatory (Akbay et al. 2003) properties of the aerial parts of the plant. In addition, the chemical composition of the various extracts of nettle has been elucidated, in the aqueous extract of nettle, Güler (2013) detected the presence of caffeic acid, gallic acid, quercetin, scopoletin, carotenoids, secoisolariciresinol and anthocyanidins. Finally, according to the Algerian traditions, the infusion of nettle leaves is recommended against rheumatism, gout and asthma (Beloued 1998).

This study scientifically validates antiasthmatic traditional use of the nettle. The effect of the traditional extract on murine experimental asthma model was studied. The murine model of OVA-induced asthma allows us to understand this disease, to evaluate the role of oxidative stress and to study the therapeutic effect of local medicinal plant.

Ovalbumin (OVA) derived from chicken egg is a frequently used allergen that induces a robust, allergic pulmonary inflammation in laboratory rodents. This animal model features many similarities to human allergic asthma, including the presence of eosinophilic lung inflammation, the release of inflammatory mediators and cytokines, and the presence of airway hyperresponsiveness after the antigen-challenge. Total and differential cell counts of inflammatory cells are performed on the bronchoalveolar lavage fluid (BALF) and serum to observe the time course of inflammation and evaluate the effects of nettle extract.

As we studied, the phytochemical composition of the extract to highlight some of antioxidant molecules (polyphenols) may reduce oxidative stress which can contribute to the exacerbation of bronchial inflammation.

## Materials and methods

### Plant material and chemicals

Fresh entire leaves of *U. dioica* (UD) were collected in March 2013 from East flank of Mount Edough (facing the Mediterranean Sea) in Sidi Aissa. This semi-urban area, located in the north of the conurbation of Annaba (36° 55–30.84 N/7° 44–52.56 E) is weakly urbanized with very low traffic. Harvesting was carried out according to good harvesting practices for medicinal plants established by OMS (2003). The plant was identified by A CHEFROUR, a professor in botany at Badji Mokhtar University and listed on I May 1903 by M. Christian at the ENSA Herbarium (Algiers, Algeria).

Ammonium molybdate, gallic acid and vanillin were obtained from Biochem, Chemopharma (Cosne Sur Loire, France). Chicken egg albumin (ovalbumin, OA, grade II), bovine serum albumin (BSA) and all other chemical products used in this study were purchased from Sigma Chemical Co (St. Louis, MO).

### Extraction

The leaves were washed thoroughly under tap water, air-dried and powdered using electric grinder to obtain a powder. 10g of UD leaves powder were boiled with 200 mL of distilled water for 20 min with an occasional stirring. The decoction preparation was then filtered through a muslin cloth followed by filtration with filter paper. The extract was evaporated to one-fifth of its original volume and kept at 4 °C until its use.

### Colorimetric assays of phenolic compounds

A quantitative analysis of the phenols (Gargouri et al. 2013), flavonoids (Bouaziz et al. 2010), flavanols (Yermakov et al. 1987), tannins (Price et al. 1978) and anthocyanins (Lee et al. 2005) were performed.

### The antioxidant activity *in vitro*

The study of antioxidant activity of the extract was carried out by three methods: DPPH test (Bouaziz et al. 2008), the NBT test (Yagi et al. 2002) and antioxidant activity (Prieto et al. 1999).

### Animals

Male Wistar rats, about 160 g body weight were obtained from Pasteur Institute (Algiers, Algeria). The animals were housed for 1 week in a room with the temperature ranging from 22 to 25 °C under constant photoperiod and given free access to food and water before the experiments were performed. They were used in accordance with the guidelines of the Committee on Use of Laboratory Animals (Déclaration de Bâle 2010). All protocols used in this study were approved under the PNR/SF 08/2012 by the Ethical Committee of Directorate General for Scientific Research and Technological Development at Algerian Ministry of Higher Education and Scientific Research.

Female rats were not used to avoid any effects of the sexual cycle on immune reactions. Rats were divided into four groups and given free access to a standard laboratory diet and water during the experimental period. Group I (CTL): negative control; group II (OVA): Ovalbumin sensitized/challenged rats (positive control); group III (UD): received UD extract (1.5 g/kg/day) orally along the experimental protocol; group IV (O/UD): received UD extract (1.5 g/kg/day) orally along the experimental protocol and sensitized/challenged with ovalbumin.

### Sensitization and antigenic challenge

OVA and O/UD groups were sensitized to ovalbumin (grade II, ref. A5253-250 G, Sigma Aldrich), according to the methods of Moura et al. (2005) and Yang et al. (2011).

They were actively sensitized by i.p. injections of ovalbumin (1 mg/mL) with alum (1 mg/mL in saline) as an adjuvant on days 0 and 14. On days 21, 22 and 23, rats were challenged for 30 min with inhalation of either OVA (5 mg/mL) via a nebulizer (OMRON, NE-C29-E) coupled to a plastic box. CTL and UD groups received injections and challenged with saline only. Rats were sacrificed 24 h after the final aerosol challenge for the collection of blood, bronchoalveolar lavage fluid (BALF) and organs (liver and lungs).

### Bronchoalveolar lavage and cytologic examination

The tracheae were cannulated, and the airway lumina were washed with 3 × 2 mL of saline solution. The liquid collected BALF was centrifuged at 400 *g* for 10 min. The cell pellet was vortexed and resuspended in 500 μL of saline and for the leucocyte counting. The supernatant for its part was used for the determination of interleukin-4.

The differential cell counts were performed in cytocentrifuge preparations of BALF stained with GIEMSA. The numbers of eosinophils and lymphocytes will be expressed in percentage relative to the total leucocytes.

### Measurement of interleukin-4

Enzyme-linked immunosorbent assays (ELISAs) were performed according to the manufacturer’s directions. Interleukin-4 (IL-4) contained in BALF and serum was measured using a specific rat IL-4 ELISA kit (Invitrogen, Camarillo, CA). The concentration of IL-4 is determined by comparison to the standard curve and is expressed as pg/mL.

### Histopathological analysis

For the histopathological analysis, the lung tissues were dissected and the tissue samples were immediately fixed in formalin solution, embedded in paraffin. The paraffin sections were cut into 5 μm thick slices and stained with haematoxylin and eosin (H&E) for light microscopic examination. The sections were viewed and photographed by a compound binocular light microscope (Hould 1984).

### Tissue preparation

About 1 g of the organ (liver or lung) was homogenized in buffer solution (2 mL) of phosphate-buffered saline 1:2 (w/v; 1 g tissue with 2 mL PBS, pH 7.4). Homogenates were centrifuged at 10,000 *g* for 15 min at 4 °C. The supernatants were divided into aliquots, then stored at 20 °C, and used for the determination of malondialdehyde (MDA), reduced glutathione (GSH) levels and the activities of the antioxidant enzymes SOD, CAT and GPx.

### Estimation of lipid peroxidation

The detection of malondialdehyde (MDA) resulting from the degradation of polyunsaturated fatty acids with 3 or 4 double bonds peroxidized by a colorimetric reaction with thiobarbituric acid (TBA), is a highly sensitive method for determining lipid peroxidation *in vitro*. This assay is performed according to the method by Esterbauer et al. (1992) in liver, lung tissues and erythrocytes. Supernatant (375 μL) was homogenized by sonication with PBS (150 μL), TCA-BHT (375 μL) (trichloroacetic acid- butylhydroxytoluene) in order to precipitate proteins, and then centrifuged (1000 *g*, 10 min, and 4 °C). After that, 400 μl of supernatant were mixed with 80 μL of HCl (0.6 M) and TBA (320 μL) dissolved in Tris, and the mixture was heated at 80 °C for 10 min. The absorbance of the resultant supernatant was read at 530 nm.

### Estimation of reduced glutathione

Glutathione level is determined according to the method by Weckbercker and Cory (1988) in the liver, lung tissues and erythrocytes. The principle of this assay is based on measuring the optical absorbance of the 2-nitro-5-mercapturic resulting from the reduction of 5,5-dithio-bis-2-nitrobenzoic acid (DTNB) by groups (SH) of glutathione. To this end, deproteinization is made to keep only (SH) groups specific to glutathione. Briefly, liver supernatant (0.8 mL) was added to 0.25% sulphosalycylic acid (0.3 mL) and tubes were centrifuged at 2500 *g* for 15 min. Supernatant (0.5 mL) was mixed with 0.025 mL of 0.01 M DTNB and 1 mL phosphate buffer (0.1 M, pH 7.4). Finally, absorbance at 412 nm was recorded. Total GSH content was expressed as n mol GSH/mg protein.

### Measurement of antioxidant enzyme activities

The antioxidant enzyme activities were determined in the liver, lung tissues and in erythrocytes by measuring the enzymatic activities of glutathione peroxidase (GPx), superoxide dismutase (SOD) and catalase (CAT).

### Measurement of glutathione peroxidase activity

The enzymatic activity of GPx activity was measured by the method of Flohe and Günzler (1984). This method is based on the reduction of hydrogen peroxide (H_2_O_2_) in the presence of reduced glutathione (GSH). The latter is transformed into glutathione disulphide (GSSG) under the influence of GPx. Supernatant obtained after centrifuging 5% liver homogenate at 1500 *g* for 10 min followed by 10,000 *g* for 30 min at 4 °C was used for GPx assay. Reaction mixture (1 mL) was prepared which contained 0.3 mL of phosphate buffer (0.1 M, pH 7.4), 0.2 mL of GSH (2 mM), 0.1 mL of sodium azide (10 mM), 0.1 mL of H_2_O_2_ (1 mM) and 0.3 mL of liver supernatant. After incubation at 37 °C for 15 min, reaction was terminated by addition of 0.5 mL 5% TCA. Tubes were centrifuged at 1500 *g* for 5 min and the supernatant was collected. 0.2 mL of phosphate buffer (0.1 M, pH 7.4) and 0.7 mL of DTNB (0.4 mg/mL) were added to 0.1 mL of reaction supernatant. After mixing, absorbance was recorded at 420 nm.

### Measurement of superoxide dismutase activity

The dosage of SOD activity was carried out by the NBT test, which is a photoreduction method riboflavin complex/methionine, which generates superoxide anions. The oxidation of NBT by the superoxide anion O_2_ is used due to the presence of SOD detection base. In an aerobic environment, riboflavin blend, methionine and NBT give a bluish colouration. The presence of SOD inhibits the oxidation of NBT (Beyer & Fridovich 1987). Briefly, 5 μL of the supernatant was combined with 1 mL of EDTA/methionine (0.3 mM), 1890 mL phosphate buffer (pH 7.8), 85 μL of 2.6 mM NBT; 22 μL of riboflavin (0.26 mM) was added as the last and the light was switched. The reaction changes in absorbance at 560 nm were recorded after 20 min.

### Measurement of catalase activity

CAT activity was measured at 240 nm using a UV/visible by the variation of the consecutive optical density at the dismutation of hydrogen peroxide (H_2_O_2_) (Aebi 1984). The reaction mixture consists of 780 μL phosphate buffer (pH 7.5), 200 μL of hydrogen peroxide (500 mM) and 20 μL supernatant in a final volume of 1 mL. Absorbance was recorded at 240 nm every 15 s for 1 min. The enzyme activity was calculated by using an extinction coefficient of 0.043 mM cm^−1^.

### Statistical analysis

Data were expressed as mean ± standard error (SE). The calculations were performed using MINITAB software (Version17) and Microsoft Excel (2007) analysis and statistical processing of data. *p* Values <0.05 were considered statistically significant.

## Results and discussion

### Phenolic composition and antioxidant activity of the UD extract

Our choice of the *U. dioica* was due to the fact that it is a local widespread medicinal plant, whose traditional uses in several pathologies are extensively documented. Table 1 shows the amount of phenols in nettle extract. The amount of flavonoids is in agreement with that given by Güler (2013), but the polyphenols concentration is more to less low compared to the results by Kaledaite and Bernatoniene (2011). The differences in the contents recorded are probably due to the influence of climate because the synthesis of polyphenols, despite its dependence on genetic factors, it is particularly sensitive to environmental conditions, including changes in the water status, the period of maturation, temperature and radiation (Husain & Shah 2011).

**Table 1.** Polyphenols, flavonoids, flavanols, tannins and anthocyanins content and scavenging capacity of *U. dioica* extract.

			Total antioxidant activity						
Samples	DPPH IC_50_ (μg/mL)	NBT IC_50_ (μg/mL)	BHT (mg BHT/mg extract)	Vit C (mg vit C/mg extract)	Total polyphenols (mg GA/g DE)	Flavonoids (mg Q/g DE)	Flavanols (mg R/g DE)	Tannins (mg C/g DE)	Anthocyanins (mgC-3G/gDE)
UD extract	152.34 ± 0.37	353.15 ± 0.70	0.876 ± 0.03	0.582 ± 0.04	30.79 ± 0.96	22.58 ± 1.02	6.30 ± 1.59	1.05 ± 0.34	0.17 ± 0.03
BHT	22.5 ± 0.62	189.27 ± 6.23	__	__	__	__	__	__	__
Vit C	3.14 ± 0.36	167.47 ± 1.40	__	__	__	__	__	__	__

Values are mean ± SE of three replicates for each estimation.

Therefore, the antioxidant activity of plant extracts is usually linked to their phenolic content. The comparison of the three tests shows that the aqueous extract of *U. dioica* has a low antioxidant activity compared with the two positive controls (Table 1). But, our extract is more active against DPPH radical than the aqueous extract of Güler (2013) work. In fact, environmental conditions and the differential geographical distribution, which can change the constitution of plant on phenolic compounds and their derivatives (phenolic acids, flavonoids, etc.), also induces differences in their antioxidant power (Husain & Shah 2011) and suggests its use as a potential source of bioactive compounds, including antioxidant activity.

### Effect of UD extract on airway inflammation

The results obtained when treating rats with the extract was compared firstly with the control group (CTL) and secondly with the OVA group (OA-sensitized rats), concerning body and relative weights, the cellular influx into the lungs and the concentration of inflammatory mediators.

In the present experiment, no significant change in body weight was recorded, which is in accordance with the work by Mahajan and Mehta (2011). However, Table 2 shows a decrease of 2.82% of body weight in rats sensitized to OA compared to the control group and 2.04 g weight gain for the rats treated with nettle (UD). This result is in agreement with that obtained by Juma et al. (2015), who noticed a weight gain of 1.27 g during treatment with an aqueous extract of nettle. Furthermore, in the present work, the obtained results showed gain in body weights of rats sensitized to OA and treated with nettle (4.21%) compared with the rats only sensitized to OA, suggesting that the administration of the UD extract has a protective effect (Table 2).

**Table 2.** Body weights of rats and relative weights of lungs and liver.

	CTL	OVA	UD	O/UD
Initial BW of rats	164.17 ± 2.77	165.57 ± 2.62	164.29 ± 1.86	164.71 ± 3.62
Final BW of rats	281.66 ± 4.41	284.28 ± 4.12	287 ± 4.69	285 ± 6.24
Weight gain	+117.49	+108.71	+122.71	+120.29
BW of rats compared to T (%)	–	−2.82	2.04	1.27
BW of rats compared to OVA (%)	–	–	–	4.21
RW of lungs (g)	0.74 ± 0.13	0.91 ± 0.13	0.76 ± 0.06	0.78 ± 0.04
RW of lungs compared to T (%)	–	22.97	2.70	5.40
RW of lungs compared to OVA (%)	–	–	–	−14.28
RW of liver (g)	3.38 ± 0.13	3.91 ± 0.17	3.44 ± 0.24	3.83 ± 0.27
RW of liver compared to T (%)	–	15.68	1.77	13.01
RW of liver compared to OVA (%)	–	–	–	−2.30

BW: body weights; RW: relative weight.

Regarding the effect on relative organ weight, the largest variation can be confirmed by the increase in the relative weight of the liver (15.68%) and lungs (22.97%) in OA sensitized rats compared with control rats (Table 2). In fact, Mauser et al. (2013) suggested that the provocation with the allergen (ovalbumin, among others) induced increased microvascular infiltration and oedema, thus the swelling of the inflamed organ. On the other hand, the relative weight of lungs decreased from the lot sensitized and treated with nettle O/UD (−14.28%) compared to the lot sensitized to OVA. This also suggests that the use of the extract decreases the tissue damage and oedema in the lungs which would have been the cause of increasing their weight.

This inflammatory phenomenon, which is due to the penetration of the allergen, results in the ‘diapedesis’: the migration of inflammatory cells from the vascular compartment to the lungs. Indeed, upon the inhalation of an allergen, the relative proportion of some cells (mainly leukocytes) located at blood circulation or respiratory tract was modified.

**Figure 1.**
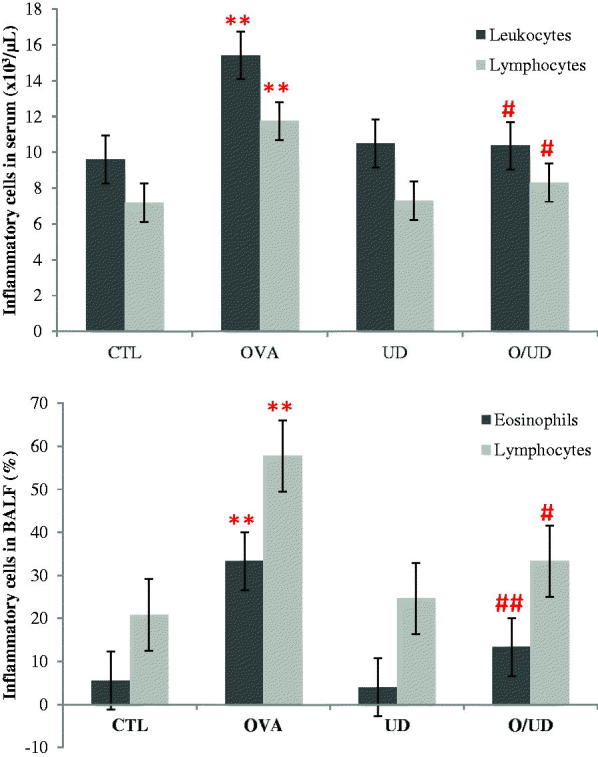
Effect of UD extract on inflammatory cells in serum and BALF. **: *p* values < 0.01 were considered statistically significant compared to control rats; #: *p* values < 0.05 were considered statistically significant compared to OA sensitized rats; ##: *p* values < 0.01 were considered statistically significant compared to OA sensitized rats.

Thus, the migration of inflammatory cells, in particular eosinophils in the lungs, by chemotaxis and leading to their diapedesis, mainly contributes to the development of airway inflammation (Yuk et al. 2007). The infiltration of eosinophils is one of the principal characteristics of allergic inflammation associated with bronchial hyperresponsiveness and this by the release of various proteins and ROS. Moreover, it has been proven that it is the activated eosinophils that induce epithelial damage characteristics in asthmatic subjects (Raju et al. 2014). Thus, in the OA-sensitized rats, a significant (*p* < 0.01) increase in the rate of eosinophils in BALF compared to the control group was clearly seen, confirming the presence of a recruitment of eosinophil and their extravasation towards lungs. These results shown in Figure 1 are in agreement with Yuk et al. (2007) and Lee et al. (2010b).

Similarly, in the present study, the rats exposed to ovalbumin (OVA lot) showed a significant (*p* < 0.01) increase in the rate of leucocytes compared to control rats (Figure 1), confirming the presence of an inflammatory state as it was already described in the Xiang-hua et al. (2009) work.

On the other hand, a significant decrease (*p* < 0.05) in serum leucocyte level was noted between rats sensitized with OA and treated with the extract (O/UD) and rats only sensitized to OA (OVA). Similarly, the rate of eosinophils decreased significantly (*p* < 0.01) in BALF during the use of nettle extract in the O/UD lot compared to OVA lot. The treatment with nettle has reduced the inflammatory condition by reducing leucocytes rates to their normal value and decreasing the recruitment of eosinophils in the lungs. This suggests that nettle would improve the defence mechanisms of the body as it has been reported in the literature (Juma et al. 2015).

Among leucocytes, lymphocytes are important regulators of the immune response and their role in asthma is related to chronic inflammation. In addition, several studies on rats and guinea pigs, in which experimental asthma was caused by exposure to ovalbumin, have shown an increase in lymphocyte ratio (Xiang-hua et al. 2009; Pankaj et al. 2011). This corresponds well with our results, confirmed with a significant increase (*p* < 0.01) in the lymphocyte levels in the OVA lot compared to the control group in serum and BALF. Indeed, a significant decrease (*p* < 0.05) in lymphocytes level can be observed in both serum and BALF in O/UD lot compared to OVA lot. These decreases are due to a decrease in the recruitment of lymphocytes, once again claiming the reduction of lung inflammation (Figure 1).

**Figure 2.**
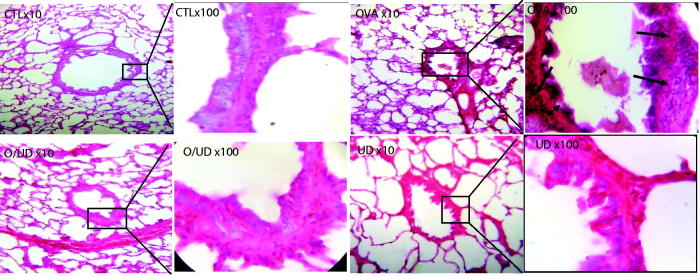
Effect of UD extract on the recruitment of leucocytes in lung tissue. Histological examination of lung tissue was performed 48 h after the final OVA challenge. (Magnification 10× et 100×).

**Figure 3.**
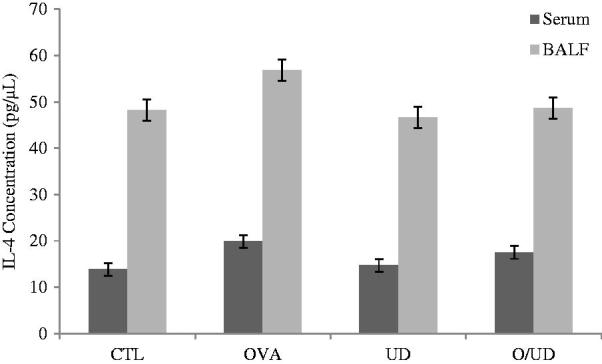
Effect of UD extract on Il-4 concentration in serum and BALF.

In fact, during the asthmatic inflammation, cells infiltrating the lungs, such as eosinophils and lymphocytes secrete Th2-type cytokines (Yuk et al. 2007), including IL-5, IL -13 and IL-4. IL-4 is particularly important as it acts as a growth factor for Th2 cells and promotes the migration of eosinophils into lung tissue, adhesion to endothelial cells and mucus production. It is also the primary mediator of immunoglobulin class switching (Ig) that induces the human expression of low affinity receptor, FcɛRII, for IgE (Belleau et al. 2005). In addition, the increased levels of these cytokines stimulate the profibrotic transformation of TGF-β, which is expressed in various lung cells such as alveolar macrophages, fibroblasts and endothelial cells by inducing increased accumulation of the extracellular matrix and thickening of the inter- alveolar septa (Halwani et al. 2011). Indeed, these structural and architectural changes were observed in the bronchi of OA-sensitized rats (OVA group) (Figure 2). The lungs seem to be infiltrated with inflammatory cells with a thick epithelium, oedema and a few centres of haemorrhage. The presence of mucus and inflammatory cells in the lumen of bronchioles was also observed, which is a widely described phenomenon in the literature leading to airway remodelling (Ra et al. 2010). Furthermore, our study provides evidence to the anti-inflammatory and anti-asthmatic effect of the nettle extract, since the histological observation of rat lung tissues of the O/UD lot showed a decrease in the influx of inflammatory cells to the lungs as well as in the signs of inflammation and airway obstruction (Figure 2). Similarly, no histological changes in the lungs of UD lot compared to the control group were found. Moreover, the treatment of sensitized rats with nettle extract decreases the concentration of IL-4 to 12.05% in serum and 14.36% in the BALF, respectively (Figure 3). A similar concept was found in the literature, demonstrating the inhibitory role of the leaf extract of *U. dioica* in the production of the proinflammatory transcription factor NF-kB (Riehemann et al. 1999), which was attributed by Kandasamy et al. (2012) to the anti-asthmatic activity of flavonoids. In fact, several other studies on bioactive molecules have suggested that chrysin reduces the infiltration of inflammatory cells in a murine asthma model by restoring the balance Th1/Th2 (Pankaj et al. 2011). As a result, the treatment with the extract of nettle having induced IL-4 concentration reduction may be associated with a restoration of the balance between Th1 and Th2 cytokines. This is likely to be caused by its content in flavonoids as it has been reported by Güler (2013) and Kandasamy et al. (2012). Actually, in the present study, it is reported that our nettle extract was not only rich in phenolic compounds, but also had a significant antioxidant potential, hence the importance to complete our investigation by studying its effect on oxidative stress generated by the inhalation of the allergen, and participates in turn exacerbate bronchial inflammation.

### Effect of UD extract on oxidative stress induced by ovalbumin sensitized

To the best of our knowledge, the present research work is pioneering in determining the antioxidant and anti-inflammatory effects of our aqueous extract of UD in the case of the asthmatic pathology. Indeed, a few studies have indicated that supplementation of *U. dioica* has promoted antioxidant defence systems and decreased lipid peroxidation (Yener et al. 2008).

In fact, this study has proven that the nettle extract maintains the antioxidant defence systems and does not affect lipid peroxidation, since there is no significant variation in the UD lot compared to the naïve control. However, significantly increased (*p* < 0.05) MDA levels were observed in rats sensitized in the erythrocytes and studied organs (liver and lungs) attesting to the occurrence of cell membranes damages of various tissues involved as it has been reported by Chekchaki et al. (2017) who have described an anti-inflammatory and antioxidant effects of two extracts of *Pistacia lentiscus* in the liver and erythrocytes, in an experimental model of asthma. These alterations due to the exposition to ovalbumin and accompanied by increased lipid peroxidation have been reported in the literature as the result of the penetration of the allergen in asthma patients (Tiwari et al. 2014; You et al. 2014). The presence of MDA in lung tissue may increase the chemotaxis of leucocytes. Conversely, the release of large quantities of superoxide anion and hydrogen peroxide by activated inflammatory cells in the pulmonary alveoli is the cause of MDA elevation, thus perpetuating inflammation (You et al. 2014).

**Figure 4.**
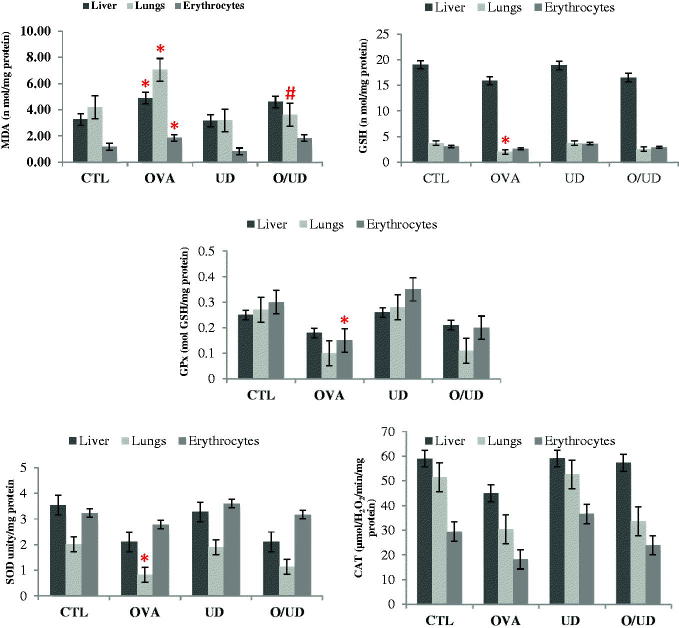
Effect of UD extract on markers of oxidative stress in liver, lungs and erythrocyte. *: *p* values < 0.05 were considered statistically significant compared to control rats ; #: *p* values < 0.05 were considered statistically significant compared to OA sensitized rats.

Therefore, the treatment of rats exposed to OA with nettle extract (O/UD lot) has reduced the MDA levels compared to OA-sensitized rats (OVA), thus reducing lipid peroxidation in the liver, erythrocytes and significantly (*p* < 0.05) in the lungs (Figure 4). This may be due to the protective effects of antioxidant molecules, found in the extract, against cell membranes damages caused by free radicals. Other studies have also shown that the treatment with certain medicinal plants and various antioxidants helps in reducing lipid peroxidation in cases of experimental asthma (Tiwari et al. 2014; You et al. 2014).

Moreover, in the asthmatic pathology, as soon as the state of stress settles, the body involves several defence systems as the glutathione system and enzymatic antioxidants such as GPx, SOD and CAT whose activities were investigated in our study. In fact, a significant decrease was recorded (*p* < 0.05) in the GSH level in the OVA lot compared to control lot (CTL) in the lungs (Figure 4). This result is in agreement with those of You et al. (2014), and those of Al-Afaleg et al. (2011). Similarly, significant (*p* < 0.05) decreases of GPx and SOD activities in the lungs were recorded in the OA-sensitized rats compared to the control rats. Indeed, several studies have shown a decrease in the GPx activity in serum and erythrocytes of patients with asthma (Mak et al. 2004; Al-Afaleg et al. 2011). Other studies have also observed a decrease in SOD activity in asthmatic patients’ lungs (Mak et al. 2004). Yet, no significant change in the activity of catalase between OA-sensitized rats and control is recorded. As noted by Mak et al. (2004), this may be due to the fact that the hydrogen peroxide formed after the dismutation of O_2_ by SOD has been removed by normal levels of catalase and high levels of glutathione, as it represents the main defender against the H_2_O_2_ toxicity. Furthermore, although the treatment of rats exposed to ovalbumin with UD extract (Lot O/UD) significantly decreases lipid peroxidation, it is not involved in the significant increase of the levels of antioxidant defence system (Figure 4), even if there were increases of 27.78% for GSH level and 10%, 39.02% and 10.87% for the activities of SOD, GPx and CAT, respectively, in the lungs.

On the other hand, according to Table 3, the studied inflammation markers are positively correlated with MDA and negatively with the remaining studied stress markers (GSH, GPx, SOD, and CAT). Thus, the increased bronchial inflammation parameter is accompanied by the increase in MDA, marker of lipid peroxidation, and decrease in GSH and antioxidant enzymes (GPx, SOD, and CAT). Indeed, when airway inflammation is increasing and therefore the recruitment of inflammatory cells increases, it is accompanied by increased levels of ROS, causing oxidative stress. This may explain the high lipid peroxidation and antioxidants decrease such as GSH, GPx, SOD, and CAT.

**Table 3.** Correlations between markers of airway inflammation and markers of oxidative stress.

Markers of oxidative stress	Correlation coefficient Pearson (*R*)				
Markers of airway inflammation	MDA	GSH	GPx	SOD	CAT
Blood					
Leucocytes	0.77	−0.94*	−0.82	−0.95*	−0.68
Lymphocytes	0.83	−0.91	−0.88	−0.97**	−0.76
IL-4	0.86	−0.86	−0.92	−0.96*	−0.77
BALF					
Eosinophils	0.61	−0.93	−0.70	−0.86	−0.50
Lymphocytes	0.77	−0.97*	−0.79	−0.97*	−0.71
IL-4	0.47	−0.95*	−0.56	−0.80	−0.38

*p values < 0.05 were considered statistically significant compared to control rats. **p values < 0.01 were considered statistically significant compared to control rats.

## Conclusions

The treatment of rats sensitized to ovalbumin with *U. dioica* extract, traditionally used against asthma and inflammation, was proven to be effective in relieving pulmonary inflammation through the significant reduction of level and infiltration of inflammatory cell in lungs. Indeed, at the sites of inflammation, inflammatory cells such as eosinophils generate ROS that contribute to tissue damage and inflammatory reactions. In addition, lung epithelial cells are known to produce ROS in response to various environmental stimuli. These ROS oxidize membrane phospholipids and were found to induce alterations in its functions such as transportation and loss of its fluidity.

Hence, the regulation of ROS rates during inflammation would contribute to the treatment of inflammatory lung diseases. Our results have shown a significant decrease in inflammatory cell recruitment and lipid peroxidation during the use of the nettle extract (Lot O/UD) compared to the OVA lot, in which the increase was significant.

This study is ultimately a piece of evidence for the traditional therapeutic indications of the extract of nettle (*U. dioica)* in the asthmatic airway inflammation.

## References

[CIT0001] Aebi H. 1984 Catalase *in vitro*. Meth Enzymol. 105:121–126. 6727660 10.1016/s0076-6879(84)05016-3

[CIT0002] Akbay P, Basaran AA, Undeger U, Basaran N. 2003 *In vitro* immunomodulatory activity of flavonoid glycosides from *Urtica dioica* L. Phytother Res. 17:34–37. 12557244 10.1002/ptr.1068

[CIT0003] Al-Afaleg NO, Al-Senaidy A, El-Ansary A. 2011 Oxidative stress and antioxidant status in Saudi asthmatic patients. Clin Biochem. 44:612–617. 21320478 10.1016/j.clinbiochem.2011.01.016

[CIT0004] Belleau JT, Gandhi RK, Pherson MHM, Lew DB. 2005 Research upregulation of CD23 (Fc epsilon RII) expression in human airway smooth muscle cells (huASMC) in response to IL-4, GM-CSF, and IL-4/GM-CSF. Clin Mol Allergy. 3:6. 15907205 10.1186/1476-7961-3-6 PMC1173127

[CIT0005] Beloued A. 1998 Medicinal plants in Algeria. Alger: Office of University Publications, p. 62.

[CIT0006] Beyer WF, Fridovich I. 1987 Assaying for superoxide dismutase activity: some large consequences of minor changes in conditions. Anal Biochem. 161:559–566. 3034103 10.1016/0003-2697(87)90489-1

[CIT0007] Bouaziz M, Feki I, Ayadi M, Jemai H, Sayadi S. 2010 Stability of refined olive oil and olive pomace oil added by phenolic compounds from olive leaves. Eur J Lipid Sci Technol. 112:21–24.

[CIT0008] Bouaziz M, Feki I, Jemai H, Ayadi M, Sayadi S. 2008 Effect of storage on refined and husk olive oils composition: stabilization by addition of natural antioxidants from chemlali olive leaves. Food Chem. 108:253–262.

[CIT0009] Chekchaki N, Khaldi T, Rouibah Z, Rouag M, Sekiou O, Messarah M, Boumendjel A. 2017 Anti-inflammatory and antioxidant effects of two extracts from *Pistacia lentiscus* in liver and erythrocytes, in an experimental model of asthma. Int J Pharm Sci Rev Res. 42:77–84.

[CIT0010] Chrubasik S, Enderlein W, Bauer R, Grabner W. 1997 Evidence for anti-rheumatic effectiveness of Herba *Urtica dioica* III acute arthritis: a pilot study. Phytomedicine. 1:105–108. 10.1016/S0944-7113(97)80052-9 23195396

[CIT0011] Déclaration de Bâle 2010. Appel à plus de confiance, de transparence et d’échanges au sujet de la recherche sur les animaux. La première conférence de Bâle, La recherche à la croisée des chemins.

[CIT0012] Dworsky R. 2000 Oxidant stress in asthma. Thorax. 55:S51–S53. 10.1136/thorax.55.suppl_2.S51 PMC1765968 10992559

[CIT0013] Esterbauer H, Gebicki J, Puhl H, Jungens G. 1992 The role of lipid peroxidation and antioxidants in oxidative modification of LDL. Free Radic Biol Med. 13:341–390. 1398217 10.1016/0891-5849(92)90181-f

[CIT0014] Flohe L, Günzler WA. 1984 Analysis of glutathione peroxidase. Meth Enzymol. 105:114–121. 6727659 10.1016/s0076-6879(84)05015-1

[CIT0015] Gargouri B, Ammar S, Zribi A, Ben Mansour A, Bouaziz M. 2013 Effect of growing region on quality characteristics and phenolic compounds of chemlali extra-virgin olive oils. Acta Physiol Plant. 35:2801–2812.

[CIT0016] Güler ER. 2013 Investigation of chemopreventive properties of *Urtica dioica* L., in MCF-7 and MDA 231 breast cancer cell lines. New J Med. 30:50–53.

[CIT0017] Halwani R, Al-Muhsen S, Al-Jahdali H, Hamid Q. 2011 Role of TGF-β in airway remodeling in asthma. Am J Resp Cell Mol Biol. 44:12–133. 10.1165/rcmb.2010-0027TR 20525803

[CIT0018] Hould R. 1984 Technical on histopathology and cytopathology. Maloine. 19-21:225–227.

[CIT0019] Husain MA, Shah MDA. 2011 Study on the total phenols content and antioxidant activity of essential oil and different solvent extracts of endemic plant *Merremia borneensis*. Arab J Chem. 8:66–71.

[CIT0020] Juma KK, Maina SG, Muriithi JN, Mwangi BM, Mworia JK, Mwonjoria MJ, Ngeranwa JJN, Mburu DN. 2015 Protective effects of *Urtica dioica* and cimetidine on liver function following acetaminophen induced hepatotoxicity in mice. J Develop Drugs. 4:2.

[CIT0021] Kaledaite R, Bernatoniene J. 2011 Investigation of antiradical activity of *Salvia officinalis* L., *Urtica dioica* L., and *Thymus vulgaris* L. extracts as potential candidates for a complex therapeutic preparation. J Med Plant Res. 5:6090–6096.

[CIT0022] Kandasamy RGR, Hellermann SS, Mohapatra RF, Lockey A. 2012 Flavonoid-rich alcoholic extract of leaves of *Achyranthes aspera* reduces inflammation in a murine model of ova-induced asthma. J Allergy Clin Immunol. 129:AB78.

[CIT0023] Kavalali GM. 2003 *Urtica*: therapeutic and nutritional aspects of stinging nettles In: Hardman R., Editor. Medicinal and aromatic plants – industrial profiles. vol. 37 London: Taylor & Francis Ltd, p. 47–55.

[CIT0024] Lee J, Durst RW, Wrolstad RE. 2005 Determination of total monomeric anthocyanin pigment content of fruit juices, beverages, natural colorants, and wines by the pH differential method: Collaborative study. J AOAC Int. 88:1269–1278. 16385975

[CIT0025] Lee MY, Lee NH, Seo CS, Lee JA, Jung D, Kim JH, Shin HK. 2010a *Alpinia katsumadai* seed extract attenuate oxidative stress and asthmatic activity in a mouse model of allergic asthma. Food Chem Toxicol. 48:1746–1752. 20385191 10.1016/j.fct.2010.04.004

[CIT0026] Lee NM, Lee MY, Lee JA, Jung DY, Seo CS, Kim JH, Shin HK. 2010b Anti-asthmatic effect of *Sanguisorba officinalis* L. and potential role of heme oxygenase-1 in an ovalbumin-induced murine asthma model. Int J Mol Med. 26:201–208. 20596599 10.3892/ijmm_00000453

[CIT0027] Mahajan SG, Mehta AA. 2011 Suppression of ovalbumin-induced Th2-driven airway inflammation by β-sitosterol in a guinea pig model of asthma. Eur J Pharmacol. 650:458–464. 20946894 10.1016/j.ejphar.2010.09.075

[CIT0028] Mak JCW, Leung HCM, Ho SP, Law BK, Lam WK. 2004 Systemic oxidative and antioxidative status in Chinese patients with asthma. J Allergy Clin Immunol. 114:260–266. 15316500 10.1016/j.jaci.2004.05.013

[CIT0029] Mauser PJ, House H, Jones H, Correll G, Boyce C, Chapman RW. 2013 Pharmacological characterization of the late phase reduction in lung functions and correlations with microvascular leakage and lung edema in allergen-challenged Brown Norway rats. Pulm Pharmacol Therap. 26:677–684. 23523662 10.1016/j.pupt.2013.03.005

[CIT0030] Mittman P. 1990 Randomized, double-blind study of freeze-dried *Urtica dioica* in the treatment of allergic rhinitis. Planta Med. 56:44–47. 2192379 10.1055/s-2006-960881

[CIT0031] Moura CTM, Bezerra FC, Moraes IM, Magalhães PJC, Capaz FR. 2005 Increase responsiveness to 5-hydroxytryptamine after antigenic challenge is inhibited by nifedipine and niflumic acid in rat trachea *in vitro*. Clin Exp Pharmacol Physiol. 32:1119–1123. 16445579 10.1111/j.1440-1681.2005.04308.x

[CIT0032] OMS 2003. OMS guidelines on good agricultural practices and good harvesting practices, for medicinal plants (BPAR), 84.

[CIT0033] Pankaj G, Wadibhasme M, Ghaisas MM, Prasad A. 2011 Thakurdesai anti-asthmatic potential of chrysin on ovalbumin-induced bronchoalveolar hyperresponsiveness in rats. Pharm Biol. 49:508–515. 21501099 10.3109/13880209.2010.521754

[CIT0034] Price ML, Van Scoyoc S, Butler LGJ. 1978 A critical evaluation of the vanillic reaction as an assay for tannin in sorghum grain. Agric Food Chem. 26:13–12.

[CIT0035] Prieto P, Pineda M, Aguilar M. 1999 Spectrophotometric quantitation of antioxidant capacity through the formation of a phosphomolybdenum complex: specific application to the determination of vitamin E. Anal Biochem. 269:337–341. 10222007 10.1006/abio.1999.4019

[CIT0036] Ra J, Lee S, Kim HJ, Jang YP, Ahn H, Kim J. 2010 *Bambusae Caulis* in Taeniam extract reduces ovalbumin-induced airway inflammation and T helper 2 responses in mice. J Ethnopharmacol. 128:241–247. 20079411 10.1016/j.jep.2010.01.023

[CIT0037] Raju S, Kumar MNS, Gupta S, Srinivas T, Naga ST, Shankar JK, Murthy V, Madhunapanthula SR, Mulukutla S, Ambhore NS. 2014 5-Aminosalicylic acid attenuates allergen-induced airway inflammation and oxidative stress in asthma. Pulm Pharmacol Therap. 29:209–216. 25101553 10.1016/j.pupt.2014.07.007

[CIT0038] Randall CF, Randall H, Dobbs F, Hutton C, Sanders H. 2000 Randomized controlled trial of nettle sting for treatment of base-of-thumb pain. J R Soc Med. 93:305–309. 10911825 10.1177/014107680009300607 PMC1298033

[CIT0039] Riehemann K, Behnkec B, Schulze-Ostho K. 1999 Plant extracts from stinging nettle (*Urtica dioica*), an anti-rheumatic remedy, inhibit the proinflammatory transcription factor NF-KappaB. Letters. 442:89–94. 10.1016/s0014-5793(98)01622-6 9923611

[CIT0040] Tiwari M, Dwivedi UN, Kakkar P. 2014 *Tinospora cordifolia* extract modulates COX-2, iNOS, ICAM-1, pro-inflammatory cytokines and redox status in murine model of asthma. J Ethnopharmacol. 153:326–337. 24556222 10.1016/j.jep.2014.01.031

[CIT0041] Weckbercker G, Cory JG. 1988 Ribonucleotide reductase activity and growth of glutathione depended mouse Leukemia L1210 cells *in vitro*. Cancer Lett. 40:257–264. 3289734 10.1016/0304-3835(88)90084-5

[CIT0042] Xiang-hua L, Xian-yu T, Zhang D, Xu J-F, Wang W-Y, Wang WY, Zhang Y, Du YM. 2009 Effects of Wuwei Dilong Decoction on inflammatory cells and cytokines in asthma model guinea pigs. J Tradit Chin Med. 29:220–223. 19894390 10.1016/s0254-6272(09)60070-4

[CIT0043] Yagi A, Kabash A, Okamura N, Haraguchi H, Moustafa SM, Khalifa TI. 2002 Antioxidant, free radical scavenging and anti-inflammatory effects of aloesin derivatives in *Aloe vera*. Planta Med. 68:957–960. 12451482 10.1055/s-2002-35666

[CIT0044] Yang EJ, Lee J-S, Song BB, Yun C-Y, Kim D-H, Kim IS. 2011 Anti-inflammatory effects of ethanolic extract from *Lagerstroemia indica* on airway inflammation in mice. J Ethnopharmacol. 136:422–427. 20600763 10.1016/j.jep.2010.05.066

[CIT0045] Yener Z, Celik I, Ilhan F, Bal R. 2008 Effects of *Urtica dioica* L. seed on lipid peroxidation, antioxidants and liver pathology in aflatoxin-induced tissue injury in rats. Food Chem Toxicol. 47:418–424. 19073231 10.1016/j.fct.2008.11.031

[CIT0046] Yermakov AL, Arasimov VV, Yarosh NP. 1987 Methods of biochemical analysis of plants. New York, USA: John Wiley and Sons, p. 300.

[CIT0047] You H, Chen S, Mao L, Li B, Yuan Y, Li R, Yang X. 2014 The adjuvant effect induced by di-(2-ethylhexyl) phthalate (DEHP) is mediated through oxidative stress in a mouse model of asthma. Food Chem Toxicol. 71:272–281. 24953552 10.1016/j.fct.2014.06.012

[CIT0048] Yuk JE, Woo J-S, Yun C-Y, Lee J-S, Kim J-H, Song GY, Yang EJ, Hur IK, Kim IS. 2007 Effects of lactose-beta-sitosterol and beta-sitosterol on ovalbumin-induced lung inflammation in actively sensitized mice. Int Immunopharmacol. 7:1517–1527. 17920528 10.1016/j.intimp.2007.07.026

